# Comment on: Effect of bariatric surgery on cancer risk: results from an emulated target trial using population-based data

**DOI:** 10.1093/bjs/znac286

**Published:** 2022-08-23

**Authors:** Kajsa Sjöholm, Markku Peltonen, Lena M S Carlsson, Magdalena Taube

**Affiliations:** Department of Molecular and Clinical Medicine, Institute of Medicine, Sahlgrenska Academy at University of Gothenburg, Gothenburg, Sweden; Department of Chronic Disease Prevention, National Institute of Health and Welfare, Helsinki, Finland; Department of Neurobiology, Care Sciences and Society, Karolinska Institutet, Solna, Sweden; Department of Molecular and Clinical Medicine, Institute of Medicine, Sahlgrenska Academy at University of Gothenburg, Gothenburg, Sweden; Department of Molecular and Clinical Medicine, Institute of Medicine, Sahlgrenska Academy at University of Gothenburg, Gothenburg, Sweden

*Dear Editor*

We read with interest the research from Lazzati *et al.*^1^, which further strengthens our knowledge on the association between bariatric surgery and reduced risk of cancer in individuals with obesity. Nevertheless, as representatives of the Swedish Obese Subjects (SOS) study group, we need to point out that the part of the discussion addressing controversial results with respect to the incidence of colorectal cancer after bariatric surgery is misleading.

We fully agree with the authors that the inclusion of an adequately matched, obese control group is of the utmost importance when assessing the effects of bariatric surgery. However, by citing a SOS report in the Discussion erroneously, this implies that the method used in the SOS study is that of standardized incidence ratios (comparing patients who had bariatric surgery with the general population), which is not the case. Furthermore, the cited paper by Anveden *et al*.^2^ concerns female cancer, but does not discuss the incidence of colorectal cancer.

Finally, we recently published a report specifically analyzing the incidence of colorectal cancer after bariatric surgery in the prospective, controlled SOS study^3^. In that report, we found no evidence that colorectal cancer is affected by bariatric surgery (adjusted hazard ratio with surgery = 0.89 (95 per cent c.i. 0.62 to 1.29; *P* = 0.551) and also extensively discuss the controversial results with respect to colorectal cancer; we also underline the importance of adequately designed studies.
